# Assessment of myocardial performance using three-dimensional echocardiography in cancer patients exposed to anthracycline-based chemotherapy and/or targeted therapy – a scoping review

**DOI:** 10.1186/s12872-026-05891-w

**Published:** 2026-05-16

**Authors:** Ananya P Raj, Krishnananda Nayak, Karthik S Udupa, Ananth Pai, Sharada Mailankody, Kanhai Rajendra Lalani

**Affiliations:** 1 Department of Cardiovascular Technology, Manipal College of Health Professions, Manipal Academy of Higher Education, Manipal, Karnataka India; 2 Department of Medical Oncology, Kasturba Medical College, Manipal, Manipal Academy of Higher Education, Manipal, Karnataka India; 3 Department of Cardiology, Kasturba Medical College, Manipal, Manipal Academy of Higher Education, Manipal, Karnataka India

**Keywords:** Three-dimensional echocardiography, Cancer therapy–related cardiac dysfunction, Cardio-oncology, Anthracycline-based chemotherapy, Targeted therapy, Myocardial strain, Scoping review

## Abstract

**Background:**

Chemotherapy and targeted therapies, including anthracycline-based regimens or their combination, have significantly improved cancer patient survival; however, they are associated with cancer therapy–related cardiac dysfunction (CTRCD), which may not be adequately detected using conventional two-dimensional echocardiography.

**Purpose:**

To identify the available evidence on the application of three-dimensional echocardiography (3DE) in the evaluation of myocardial performance in patients undergoing anthracycline-based chemotherapy and/or targeted therapy using cancer patients as a cohort study.

**Methods:**

This scoping review was performed based on the Joanna Briggs Institute (JBI) framework of conducting scoping reviews and was reported in following the Preferred Reporting Items of systematic reviews and meta-analyses extension to Scoping Reviews (PRISMA-ScR). It searched five electronic databases (MEDLINE through PubMed, Embase, Scopus, Web of Science and Cochrane Library) dating back to November 2025. Records were independently screened and data extracted by two reviewers, any disagreement in this process was resolved by discussing with one another and when required, consulting with a third reviewer. A synthesis of 5 eligible observational studies was done using a narrative approach.

**Results:**

The studies that were included involved mostly breast cancer patients who were using anthracycline-based chemotherapy as a single agent or together with the use of HER2-targeted therapy. One of the studies assessed patients with chronic myeloid leukaemia receiving long term tyrosine kinase inhibitor (TKI) therapy whose treatment had not been exposed to anthracyclines. 3D measurements were taken to evaluate the baseline and follow-up assessment. Three-dimensional strain metrics, of which global longitudinal strain (GLS), global circumferential strain (GCS), and global area strain (GAS) were reported to identify features suggestive of subclinical myocardial dysfunction prior to observable reductions in left ventricular ejection fraction (LVEF).

**Conclusion:**

Available evidence indicates that 3DE, especially the three-dimensional myocardial strain analysis, may facilitate the earlier identification of subclinical myocardial dysfunction earlier in patients undergoing anthracycline-based chemotherapy and/or targeted therapy. Nevertheless, due to the limited number of included studies and heterogeneity in its methods, these results are to be viewed with reservations and larger standardized multicenter studies are justified.

**Supplementary Information:**

The online version contains supplementary material available at 10.1186/s12872-026-05891-w.

## Key Message

**What is already known on this topic**

Cancer therapy-related cardiac dysfunction (CTRCD) is linked to anthracycline-based chemotherapy and targeted cancer therapy, such as HER2 inhibitors and tyrosine kinase inhibitors (TKIs), and can be regarded as being subclinical myocardial injury before left ventricular ejection fraction. Two-dimensional echocardiography is a commonly used method of cardiac monitoring with limited sensitivity regarding early myocardial dysfunction.

**What this study adds**

The scoping review maps the existing body of knowledge regarding the use of three-dimensional echocardiography as a tool of measuring myocardial performance in patients undergoing chemotherapy and/or targeted therapies with anthracycline. Included studies indicate that three-dimensional strain measures and especially GLS, GCS, and GAS have been reported to detect features suggestive of subclinical myocardial dysfunction earlier than changes in ejection fraction, but the amount of evidence is still scarce.

**How this study might affect research, practice or policy**

The results point to the further research on the potential of the three-dimensional echocardiography as a sensitive method of monitoring early signs of cardiotoxicity in cardio-oncology. Informative larger prospective multi-center studies and standardized imaging protocols are required to determine its daily clinical use, to inform the adoption of more sophisticated deformation imaging in the cardiac monitoring of cancer treatment guidelines.

**Supplementary Information:**

The online version contains supplementary material available at 10.1186/s12872-026-05891-w.

## Introduction

Advances in cancer treatment have significantly improved survival outcomes across a wide range of malignancies. Anthracycline-based chemotherapy remains a cornerstone in the management of many solid and hematological cancers due to its potent antitumor activity. In addition, targeted therapies including human epidermal growth factor receptor 2 (HER2) inhibitors, tyrosine kinase inhibitors (TKIs), and immune checkpoint inhibitors (ICIs) have transformed the oncology landscape by selectively modulating tumor cell proliferation and immune responses. These agents could be given sequentially or along with anthracyclines in order to maximize treatment effects, but their usage is being associated with cancer therapy related cardiac dysfunction (CTRCD) [1, 2].

Dose-dependent cardiotoxicity induced by anthracyclines is mediated by such mechanisms as myocardial damages, oxidative stress, and ventricular remodeling [3]. The combination of anthracyclines with targeted agents, particularly HER2 inhibitors and tyrosine kinase inhibitors (TKIs), increases the risk of both overt and subclinical myocardial dysfunction [4]. Notably, myocardial damage may occur during the early stages of treatment and can often be detected before significant changes in left ventricular ejection fraction (LVEF), highlighting the need for sensitive imaging modalities [4].

Cardio-oncology is an emerging interdisciplinary field of research, which concentrates on the best balance between cancer treatment and cardiovascular safety [5, 6]. Timely identification of myocardial dysfunction is crucial to implement timely cardioprotective strategies while continuing cancer treatment [7–9]. While two-dimensional echocardiography (2DE) is the most widely applied technique for cardiac assessment, it has limited sensitivity to detect subtle changes in the myocardium, and it is based on geometric principles which may limit its accuracy [10, 11]. Three-dimensional echocardiography (3DE) can offer more accurate volume measurements, more accurate deformation (similar to strain) images, and geometry-independent measures of mechanical function [12–14]. We have only recently shown that 3DE, including global and regional strain indices, is able to detect preclinical states of myocardial dysfunction in patients with preserved LVEF [15].

A range of echocardiographic parameters is used in clinical practice to assess myocardial performance, including two-dimensional global longitudinal strain (2D GLS), tissue mitral annular displacement (TMAD), and three-dimensional strain measures. Among these, two-dimensional GLS is the most widely utilized parameter for the early detection of subclinical left ventricular dysfunction and is recommended by current consensus guidelines [16]. TMAD is an easy and reproducible measure of longitudinal activity but cannot measure circumferential and radial mechanics. As opposed to this, three-dimensional echocardiography provides longitudinal, circumferential, and area strain measurements of a single volumetric dataset that avoids the geometric assumptions of the two-dimensional method and may offer a more comprehensive measure of myocardial deformation [11–14]. Cardiac magnetic resonance (CMR) imaging is regarded as the reference standard for volumetric and functional assessment and offers superior tissue characterization. However, its high cost, limited availability, longer acquisition times, and contraindications in certain patient populations restrict its use as a routine surveillance modality [17]. In this way, 3DE can be taken as a viable intermediate modality, with higher sensitivity than other classical 2DE parameters with the benefit of an echocardiographic-based approach being both accessible and cost-effective.

Nevertheless, the current evidence, though, is heterogeneous in terms of the type of cancer, treatment regimen, imaging study, and measurement of outcome, which limits the scope of direct comparability [18–20]. In this regard, the current scoping review is expected to map and characterize the existing evidence on the use of 3DE to measure myocardial performance in cancer patients undergoing anthracycline-based chemotherapy and/or targeted therapies.

## Methodology

### Review design

This scoping review was prepared using the methodological framework of scoping review proposed by Joanna Briggs Institute (JBI) [21] and was reported using the extension of Preferred Reporting Items scoping reviews (PRISMA-ScR) guidelines [22]. The PRISMA-ScR checklist completed is given as Supplementary Material. Included studies were not formally critically evaluated as this is in line with the exploratory nature of scoping reviews as advised by JBI; the findings of this review must be viewed in the context of this methodological factor.

### Rationale

The scoping review was considered to be the most suitable method due to the emergent and heterogenous character of the evidence on three-dimensional echocardiography in cardio-oncology. The variability in cancer types, treatment, imaging, and study goals among studies rules out the possibility of a quantitative synthesis: instead, a scoping review would be the best choice to provide a map of possible extents of available evidence and gap in knowledge.

### Review objectives

The major objective of this scoping review was to chart and outline the existing evidence regarding the use of three-dimensional echocardiography in determining the performance of the myocardium of cancer patients on anthracycline-based chemotherapy and/or targeted therapy. The secondary objectives were:

1. To determine the nature of those targeted therapies evaluated in combination with or alone anthracyclines via three-dimensional echocardiography-based cardiac assessment;
2. To define the three-dimensional echocardiographic measures of myocardial functioning in this population;
3. To study the clinical scenarios that used three-dimensional echocardiography, such as background monitoring, early case of cardiotoxicity, and follow-up;
4. To find gaps in evidence base on the methodological constraints and future research areas on use of three-dimensional echocardiography in management of cardiac issues associated with cancer therapies.

### Population, Concept, Context (PCC) Framework

The eligibility criteria and the review questions were structured over the PCC framework through the following:

#### Population

Cancer patients (adults, 18 years and above) that had undergone anthracycline-based chemotherapy and/or targeted therapy (tyrosine kinase, immune checkpoint, or her2 inhibitors).

#### Concept

Evaluation of the myocardial activity by three-dimensional echocardiography.

#### Context

Cardio-oncology, such as cardiotoxicity surveillance and cancer treatment-related cardiac dysfunction surveillance.

### Review questions

The main research question was as follows: What is the size and content of existing evidence on the evaluation of myocardial performance in patients with cancer undergoing anthracycline-based chemotherapy and/or targeted therapy with three-dimensional echocardiography?

The research questions that were secondary were the following ones:

1. What targeted therapies have been compared with or without anthracyclines using three-dimensional echocardiography?
2. What are the three-dimensional echocardiographic myocardial performance parameters that have been most commonly reported?
3. In what cancer treatment levels was three-dimensional echocardiography used?
4. What are the clinical uses and constraints of three-dimensional echocardiography use in this cohort?

### Eligibility criteria

#### Inclusion criteria

Studies were included if they met the following criteria:

-  Included adult cancer patients aged 18 years and above;
- Patients received anthracycline-based chemotherapy and/or targeted therapy (e.g., HER2 inhibitors, tyrosine kinase inhibitors, or immune checkpoint inhibitors), administered sequentially, concurrently, or as single-class exposure;
-  Three-dimensional echocardiography was employed as a primary or supplementary imaging modality to assess myocardial performance;
-  Original research articles, including observational studies, clinical trials, and prospective or retrospective cohort studies;
- Published in the English language;
- Published in peer-reviewed journals.

#### Exclusion criteria

Studies were excluded if they:

-  Involved paediatric populations (age < 18 years);
-  Used only two-dimensional echocardiography without any three-dimensional echocardiographic assessment;
-  Were animal or in vitro studies;
-  Were case reports, conference abstracts, editorials, letters, commentaries, or review articles.

Although immune checkpoint inhibitors were included in the search strategy and eligibility criteria, no studies meeting all inclusion criteria that specifically evaluated ICI-related cardiotoxicity using three-dimensional echocardiography were identified during the screening process. This likely reflects the limited application of 3DE in ICI-associated cardiotoxicity research to date.

### Information sources

A comprehensive literature search was conducted across the following electronic databases, selected for their relevance to cardiovascular imaging and oncology research:

- MEDLINE via PubMed.
- Embase
- Scopus
- Web of Science
- Cochrane Library

### Search strategy

The search strategy would involve the use of terms that refer to three main concepts; (1) three-dimensional echocardiography, (2) cancer therapy-related cardiac dysfunction or cardiotoxicity, and (3) anthracycline-based chemotherapy and/or targeted therapy. Free-text words can be searched in both title and abstract fields and were searched as free-text words, and controlled vocabulary can be searched as MeSH terms in MEDLINE and Emtree terms in Embase. All search strings that can be replicated in each database are given in Supplementary Table S1.

Searching in title and abstract fields as the main means of free-text searching was decided to be as specific as possible and as feasible as possible. We admit that it might have minimized search sensitivity because some pertinent studies might describe three-dimensional echocardiographic analysis in the methodology section without using a term of 3D or three-dimensional in title or abstract. On the same note, research in which cardiotoxicity was termed differently or where mixed regimens were used might not have been identified. This limitation is further discussed in the Discussion section and must be taken into account when making sense of the rather small number of studies included.

### Study selection

The search of the electronic database revealed that 108 records were found in total, including 46 results in the MEDLINE through PubMed, 23 in the Embase, 28 in the Scopus, 11 in the Web of Science, and 0 in the Cochrane Library. All entries were added to Zotero reference management software where automatic and manual deduplication was done. The total number of records removed comprised of duplicates who received a total of 83 records as compared to 25 unique records that were screened. The number of duplicates (76.9%) is relatively high, reflecting the substantial overlap in journal indexing across the selected databases. This is a common occurrence in systematic searches of biomedical literature when multiple databases with overlapping coverage are used.

Two reviewers were used to screen the title and abstract against the pre-set eligibility criteria. Any disagreements in the screening process were overcome by discussing between the two reviewers and where there was no consensus, a third reviewer was invited. The level of inter-reviewer agreement was not measured statistically through methods like Cohens kappa; nevertheless, all the inconsistencies were recorded and followed up before the entire text screening.

After screening title and abstract, 11 records were eliminated, mostly due to the fact that they lacked exposure to anthracycline-based chemotherapy or targeted therapy, or lacked three-dimensional echocardiographic analysis. The rest of the 14 records went through a complete review of text.

**Fig. 1:**
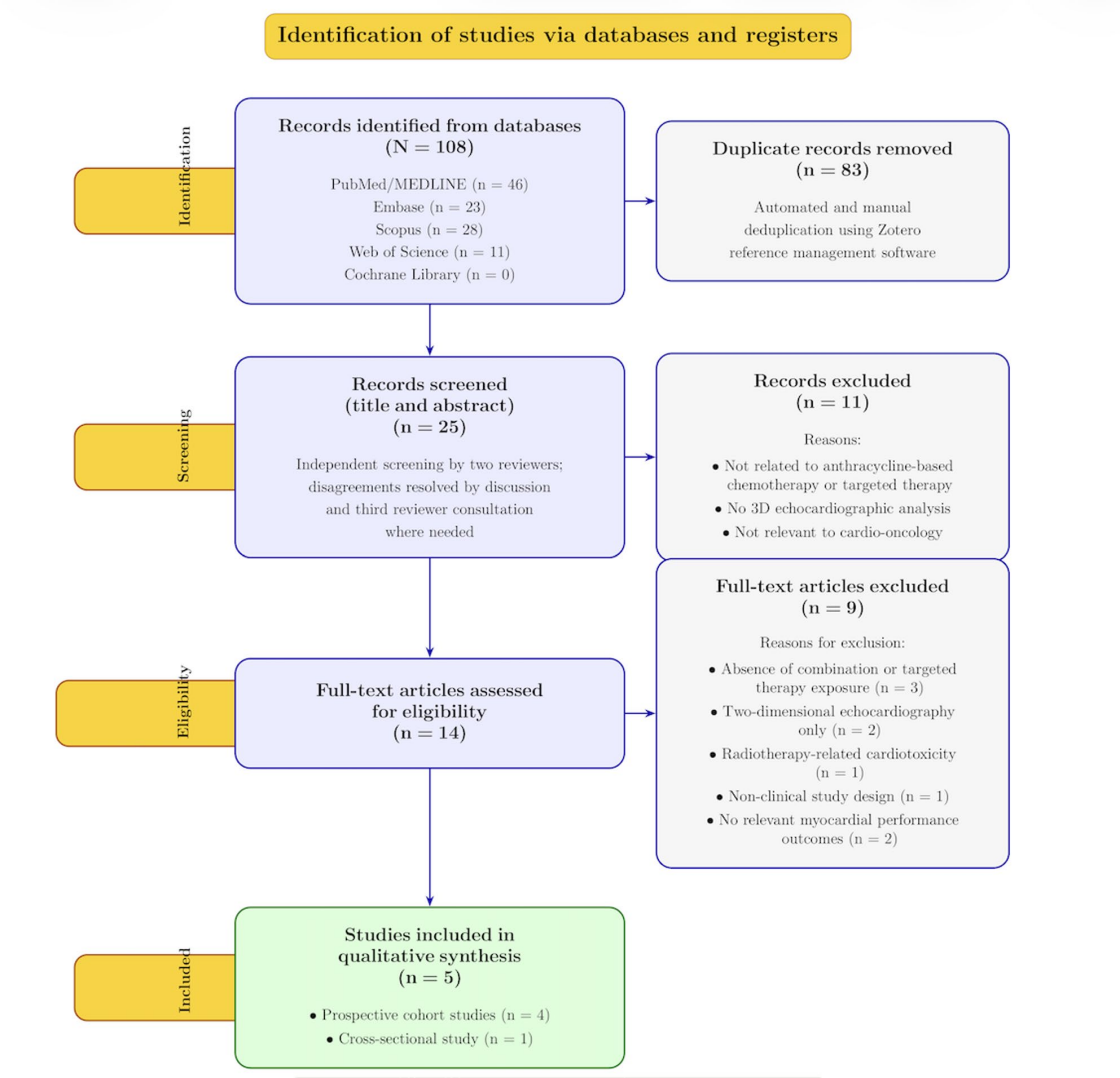
PRISMA-ScR Flow Diagram. Preferred Reporting Items for Systematic Reviews and Meta-Analyses extension for Scoping Reviews (PRISMA-ScR) flow diagram illustrating the study identification, screening, eligibility assessment, and inclusion process

The 9 studies were excluded based on the following criteria: they did not expose to combination or targeted therapy (*n* = 3), used two-dimensional echocardiography only (*n* = 2), targeted radiotherapy-related cardiotoxicity (*n* = 1), non-clinical study design (*n* = 1), and lack of appropriate myocardial performance outcomes (*n* = 2). Five articles met all the eligibility criteria and were used in the qualitative synthesis. The PRISMA-ScR flow diagram (Fig. 1) generalizes the study selection procedure and exclusion cause at every level.

### Data charting

Data were extracted independently by two reviewers using a standardised data charting form developed a priori and pilot-tested on two included studies. The following data items were extracted: study design, publication year, country, journal, cancer type, sample size, patient demographics, cancer treatment characteristics (anthracycline agents, cumulative dose, targeted therapy class and agents, treatment sequencing), three-dimensional echocardiographic parameters assessed (volumetric, functional, and deformation indices), imaging timing and clinical context, acquisition platforms and analysis software, feasibility, CTRCD definitions, and key findings related to subclinical and overt cardiac dysfunction detection. The completed data charting table is provided in Supplementary Table S2.

## Results

### Characteristics of included studies

This section provides an overview of the key characteristics of the five studies included in this scoping review, including study design, publication features, and population characteristics. Detailed study-level information is presented in Table 1.

**Table 1:** Studies excluded after full-text assessment and reasons for exclusion

No.	Study (First Author, Year)	Primary Focus	Eligibility Criterion Not Met	Reason for Exclusion
1	Xu et al., 2020 [23]	3D speckle-tracking echocardiography in breast cancer	Intervention	Evaluated myocardial performance using 3DE exclusively in patients receiving anthracycline-based chemotherapy, without exposure to targeted cancer therapy.
2	Camilli et al., 2023 [18]	LV–arterial coupling in cancer survivors	Population / Intervention	Included a paediatric population and assessed cardiac effects limited to anthracycline exposure, without evaluation of targeted-therapy-related cardiotoxicity.
3	Chen et al., 2019 [43]	Analytical chemistry (pesticide detection)	Population / Concept / Context	Non-clinical laboratory study with no human subjects, no cardiac imaging, and no relevance to cardio-oncology.
4	Santoro et al., 2017 [24]	3DE-derived strain during chemotherapy	Intervention	Assessed myocardial performance using 3DE in patients treated with anthracycline-based chemotherapy only, without targeted therapy exposure.
5	Diana et al., 2020 [39]	Echocardiographic monitoring in oncology patients	Concept / Intervention	Did not employ 3DE as the primary modality for myocardial performance assessment.
6	Lorenzini et al., 2017	Reliability of 3D LVEF	Concept	Primary objective was evaluation of observer and software variability rather than myocardial performance assessment.
7	Lu et al., 2023 [40]	3D speckle-tracking after breast cancer therapy	Intervention	Evaluated myocardial performance following anthracycline chemotherapy alone, without inclusion of targeted therapy.
8	Marwick et al., 2009/2011 [20]	Echocardiographic surveillance guidelines	Study type	Review and guideline articles without original 3DE myocardial performance data.
9	Shi et al., [41]	Myocardial function during chemotherapy	Intervention	Assessed myocardial performance exclusively in anthracycline-treated patients, without targeted therapy exposure.

Only the first 9 excluded studies are shown here for brevity. The complete list of all excluded studies with reasons is available in the supplementary materials

*Abbreviations: 3DE *three-dimensional echocardiography*, LV *left ventricular*, LVEF *left ventricular ejection fraction*, CMR *cardiovascular magnetic resonance

### Study design and publication characteristics

The five articles used observational designs; this is because the study of 3DE is exploratory research in the cardio-oncology context. Four of the studies employed prospective cohort designs [23, 25–27], whereas 1 study employed a cross-sectional observational design [28].

The articles are of a relatively recent and changing evidence base as they were published between 2019 and 2022. The rising rate of publication after 2019 is associated with the growing concern about the use of advanced echocardiographic method in early detection of cardiac dysfunction in the course of cancer treatment.

The experiments were carried out in varied geographical locations that comprised Europe (Portugal and Turkey), East Asia (China) and North America (Canada). Two were Canadian based and this implies the incorporation of cardio-oncology programmes in tertiary care centers.

### Study population characteristics

Most of the included articles were on adult cancer patients. The populations of four out of five studies were enrolled with two specifically assessing sequential anthracycline and trastuzumab in the treatment of HER2-positive breast cancer [26, 27]. A single study, originally published by Karakulak et al. (also known as Ugur et al. in earlier editions) evaluated the patients that had chronic myeloid leukaemia (CML) with long-term TKI treatment (dasatinib or nilotinib) without exposure to anthracyclines before [28]. It was added in this study since the new eligibility criteria included exposure to targeted therapy regardless of whether it was used alongside anthracycline, which allowed a wider mapping of the 3DE application in survival monitoring in cancer therapy.

The sizes of the samples were 37–136. The least was Karakulak et al. (37 patients and 50 controls) [28] and the largest cohort was Esmaeilzadeh et al. (136 patients) who had longitudinal imaging [27]. The research cohorts were mainly female due to the large number of breast cancer cohorts, and were around 40–50 s in terms of mean or median age. The CML cohort comprised of mixed sex with a larger percentage of males [28]. To reduce the confounding effects of underlying heart disease, most of the studies excluded patients who had underlying cardiovascular disease, arrhythmias, structural heart disease or low baseline LVEF.

### Cancer treatment characteristics

The cancer treatment characteristics of the included studies are summarised in Table 2.

**Table 2:** Study design and publication characteristics of included studies

Study (First Author, Year)	Study Design	Year	Country	Journal	Journal Discipline
Cruz et al., 2020 [25]	Prospective observational cohort study	2020	Portugal	Clinical Research in Cardiology	Cardiology / Cardio-oncology
Guan et al., 2021 [23]	Prospective observational cohort study	2021	China	Frontiers in Pharmacology	Pharmacology / Cardio-oncology
Lambert et al., 2020 [26]	Prospective cohort study	2020	Canada	Heart	Cardiology / Cardio-oncology
Esmaeilzadeh et al., 2022 [27]	Prospective cohort study	2022	Canada	JAMA Cardiology	Cardiology / Cardio-oncology
Karakulak et al., 2021 [28]	Cross-sectional observational study	2021	Turkey	Cardiovascular Toxicology	Cardiology / Cardio-oncology

“Ugur et al., 2020” has been corrected to “Karakulak et al., 2021 [28]” throughout, reflecting the first author of the published study [42]. The publication year has also been corrected from 2020 to 2021

### Anthracycline-based chemotherapy

Four of the five studies assessed patients exposed to anthracycline-based chemotherapy, primarily doxorubicin and epirubicin (with doses converted to doxorubicin equivalents where applicable). Only Cruz et al. reported cumulative anthracycline dose quantitatively (259.6 ± 63.3 mg/m² doxorubicin equivalent) [25]; other studies documented anthracycline exposure without specifying cumulative doses, reflecting inconsistent dose reporting practices across the literature. Treatment sequencing varied, with anthracyclines administered either concurrently with targeted therapy (Cruz et al. and Guan et al.) or sequentially followed by targeted therapy (Lambert et al. and Esmaeilzadeh et al.) [23, 25–27].

### Targeted therapy characteristics

The five studies involved the evaluation of patients undergoing targeted cancer therapy. In four clinical trials, the agent of interest was trastuzumab that was used in combination or alone in breast cancer patients who had been subjected to HER2-directed therapy. Guan et al. compared trastuzumab with or without pertuzumab with anthracycline chemotherapy [23]. The sequencing of targeted therapy relative to anthracycline exposure also varied across studies. Lambert et al. and Esmaeilzadeh et al. reported sequential administration of trastuzumab following anthracycline chemotherapy [26, 27], which is consistent with current clinical practice aimed at reducing cardiotoxicity. In contrast, Cruz et al. included patients receiving targeted therapy either concurrently with or following anthracycline exposure [25].

There was one study that did not entail exposure to anthracycline. Karakulak et al. compared patients who had undergone long-term therapy with TKI (dasatinib or nilotinib after having previously received imatinib) with a median duration of treatment duration (around 51 months) [28]. The study of cardiotoxicity in such a cohort was performed in the framework of the long-term exposure to targeted therapy with no anthracycline-related myocardial damage, which is a different paradigm of treatment.

### Three-dimensional echocardiography utilization

The utilisation of 3DE across included studies varied in terms of imaging timing, clinical context, acquisition platform, and analysis methods. These details are summarised in Tables 3, 4, 5, and 6.

**Table 3:** Study population characteristics of included studies

Study (First Author, Year)	Cancer Type	Sample Size	Mean Age (years)	Sex Distribution	Baseline Cardiovascular Risk Profile
Cruz et al., 2020 [25]	Breast cancer	105	53.8 ± 12.5	100% female	Excluded: hypertension, diabetes, CKD, prior CVD, arrhythmias, structural heart disease, baseline LVEF < 54%, cardiac medication use
Guan et al., 2021 [23]	Breast cancer	79	Median 48 (range 22–66)	100% female	Excluded: prior cardiac disease, arrhythmia; poor echocardiographic image quality
Lambert et al., 2020 [26]	HER2-positive breast cancer (early stage)	60 total (30 patients, 30 controls)	Mean ~ 50	Female (patients); mixed (controls)	Excluded: prior CVD; CMR contraindications
Esmaeilzadeh et al., 2022 [27]	HER2-positive breast cancer	136 analysed	51.1 ± 9.2	100% female	Excluded: prior anthracycline exposure, pre-existing cardiac disease; CMR contraindications
Karakulak et al., 2021 [28]	Chronic myeloid leukaemia	37 patients + 50 controls	53.5 ± 11.5	12 female / 25 male	Excluded: known CAD, heart failure, structural heart disease

*Abbreviations*: *CKD* chronic kidney disease, *CVD* cardiovascular disease, *LVEF* left ventricular ejection fraction, *CMR* cardiovascular magnetic resonance, *CAD* coronary artery disease, *HER2* human epidermal growth factor receptor 2

**Table 4:** Cancer treatment characteristics of included studies

A. Anthracycline-Based Chemotherapy								
Study (First Author, Year)		Anthracycline Agent(s)		Cumulative Dose		Dose Unit		Timing Relative to Targeted Therapy
Cruz et al., 2020 [25]		Anthracycline-based chemotherapy (doxorubicin equivalent)		259.6 ± 63.3		mg/m²		Before or alongside targeted therapy
Guan et al., 2021 [23]		Doxorubicin or Epirubicin		Not reported		Not applicable		Alone or with targeted therapy
Lambert et al., 2020 [26]		Anthracyclines (not further specified)		Not reported		Not applicable		Before targeted therapy
Esmaeilzadeh et al., 2022 [27]		Epirubicin (93%), Doxorubicin (7%)		Not reported		Not applicable		Sequential before targeted therapy
Karakulak et al., 2021 [28]		Not applicable (no anthracycline exposure)		Not applicable		Not applicable		Not applicable

B. Targeted Therapy Characteristics								
Study (First Author, Year)	Targeted Therapy Class		Specific Agent(s)		Therapy Sequencing		Treatment Approach	
Cruz et al., 2020 [25]	HER2 inhibitor		Trastuzumab (52.4% of patients)		Following or overlapping anthracycline exposure		Combination or sequential therapy	
Guan et al., 2021 [23]	HER2 inhibitors		Trastuzumab ± Pertuzumab		Concurrent with anthracycline chemotherapy		Combination therapy	
Lambert et al., 2020 [26]	HER2 inhibitor		Trastuzumab		Sequential after anthracyclines		Sequential therapy	
Esmaeilzadeh et al., 2022 [27]	HER2 inhibitor		Trastuzumab (100%)		Following anthracycline chemotherapy		Sequential therapy	
Karakulak et al., 2021 [28]	Tyrosine kinase inhibitors		Dasatinib (*n* = 17), Nilotinib (*n* = 20); prior Imatinib		Long-term TKI therapy; median duration ~ 51 months		TKI monotherapy (no anthracycline)	

*Abbreviations*: *HER2* human epidermal growth factor receptor 2, *TKI* tyrosine kinase inhibitor. Note: “Not reported” replaces previous blank cells or “Not report” entries. Karakulak et al. is correctly identified as a TKI-only study with no anthracycline exposure, consistent with the revised eligibility criteria

**Table 5:** Three-dimensional echocardiography utilization and technical characteristics

Study (First Author, Year)	Imaging Timing	Clinical Context	Echo Platform	Vendor	Analysis Software	Feasibility Reported
Cruz et al., 2020 [25]	Baseline and serial assessments during chemotherapy	On-treatment cardiotoxicity surveillance	Vivid 9 / Vivid 95	GE Healthcare	EchoPAC BT12	High overall feasibility; reduced feasibility in some basal segments
Guan et al., 2021 [23]	Baseline and after 2, 4, 6, and 8 chemotherapy cycles	Early and longitudinal cardiotoxicity monitoring	Vivid E9 / Vivid E95	GE Healthcare	EchoPAC	Feasibility reported; standardized acquisition protocol
Lambert et al., 2020 [26]	Baseline, ~ 3 months, ~6 months	On-treatment cardiotoxicity surveillance and variability assessment	Not specified	GE Healthcare	EchoPAC (echo), CVi42 (CMR)	Excellent reproducibility; feasibility implicitly high
Esmaeilzadeh et al., 2022 [27]	Baseline, post- anthracycline, 3, 6, 9, and 12 months	Early and longitudinal cardiotoxicity surveillance	Vivid E9	GE Healthcare	EchoPAC (echo), cmr42 (CMR)	≥ 91% analyzable across echocardiographic parameters
Karakulak et al., 2021 [28]	Single time point at enrolment	Detection of subclinical cardiotoxicity during long-term TKI therapy	Vivid E9	GE Healthcare	Auto LVQ	Not formally reported

*Abbreviations*: *CMR* cardiovascular magnetic resonance, *TKI*  tyrosine kinase inhibitor, *LVQ* left ventricular quantification

**Table 6:** Myocardial performance parameters and detection of cardiac dysfunction in included studies

Study (First Author, Year)	3D LV Volumes	3D LVEF	3D GLS	3D GCS	3D GAS	Regional Strain	Advanced / Additional Metrics	CTRCD Definition	Subclinical Dysfunction Detected
Cruz et al., 2020 [25]	EDV, ESV, stroke volume	Yes	Yes	Yes	Yes	Yes (17-segment LV model)	None reported	Absolute LVEF decrease > 10% to < 54% or relative GLS reduction > 15%	Yes
Guan et al., 2021 [23]	Not reported	Yes	Yes	Yes	Yes	Yes (whole LV model)	Myocardial work indices (GWI, GWE)	≥ 5% LVEF reduction to < 53% with symptoms or ≥ 10% without symptoms	Yes
Lambert et al., 2020 [26]	Not reported	Yes	Not assessed	Yes*	Not assessed	CMR-based strain analysis	CMR-derived GLS and GCS	> 10% LVEF reduction to < 55% (CMR-based)	Yes
Esmaeilzadeh et al., 2022 [27]	Not reported	Yes	Yes	Yes	Not assessed	Not performed (global strain only)	Cardiac biomarkers (hs-troponin I, BNP)	≥ 10% LVEF drop to < 55% or > 15% relative strain reduction (CMR reference)	Yes
Karakulak et al., 2021 [28]	LVEDVi, LVESVi	Yes	Yes	Yes	Yes	Not reported	None reported	Worsening myocardial strain with preserved LVEF	Yes

*Abbreviations*: *3D* three-dimensional, *LV* left ventricular, *LVEF* left ventricular ejection fraction, *EDV* end-diastolic volume, *ESV* end-systolic volume, *LVEDVi* left ventricular end-diastolic volume indexed, *LVESVi* left ventricular end-systolic volume indexed, *GLS* global longitudinal strain, *GCS* global circumferential strain, *GAS* global area strain, *GWI* global work index, *GWE* global work efficiency, *CTRCD* cancer therapy–related cardiac dysfunction, *CMR* cardiovascular magnetic resonance, *hs-troponin I *high-sensitivity troponin I, *BNP* brain natriuretic peptide

* Strain assessed using cardiac magnetic resonance rather than three-dimensional echocardiography

### Imaging timing and clinical context

All five studies included a baseline echocardiographic assessment, either before cancer treatment initiation or at the time of study enrolment, providing a reference point for subsequent myocardial performance evaluation. Four studies adopted serial imaging protocols for longitudinal monitoring. Cruz et al. performed baseline and serial echocardiographic assessments during chemotherapy for on-treatment cardiotoxicity surveillance [25]. Guan et al. employed structured follow-up assessments at 2, 4, 6, and 8 chemotherapy cycles, enabling early and progressive evaluation of myocardial function throughout treatment [23]. Lambert et al. obtained echocardiographic data at baseline and at approximately 3 and 6 months of treatment, with the dual purpose of cardiotoxicity detection and assessment of imaging variability [26]. The most comprehensive protocol was reported by Esmaeilzadeh et al., who performed baseline assessment, post-anthracycline imaging, and serial follow-up at 3-month intervals through 12 months, enabling evaluation of both early and late cardiotoxic effects [27]. Karakulak et al. employed a cross-sectional imaging strategy, obtaining 3DE data at a single time point during long-term TKI therapy, aimed at detecting subclinical myocardial dysfunction rather than longitudinal change [28].

#### Three-dimensional echocardiographic acquisition and analysis

All studies utilised three-dimensional echocardiography platforms from a single vendor, with GE Healthcare systems (Vivid series) employed consistently across cohorts, enhancing technical comparability. The majority of studies used Vivid E9 or Vivid E95 systems, while Cruz et al. additionally included earlier-generation Vivid 9 equipment [25].

For image analysis, EchoPAC software was used in all studies performing 3DE assessment, either exclusively or in conjunction with cardiovascular magnetic resonance (CMR) analysis software. Lambert et al. and Esmaeilzadeh et al. combined EchoPAC-based echocardiographic analysis with CVi42 or cmr42 software for CMR-based reference measurements, reflecting multimodality validation approaches [26, 27].

Feasibility and image quality reporting varied across studies. Cruz et al. reported high overall feasibility with reduced analysability in select basal segments [25]. Guan et al. documented feasibility within a standardised acquisition protocol [23], while Esmaeilzadeh et al. reported that at least 91% of echocardiographic parameters were analysable across imaging time points [27]. Lambert et al. did not explicitly quantify feasibility but reported excellent reproducibility, suggesting robust image quality [26]. Karakulak et al. did not formally report feasibility metrics [28].

### Myocardial performance parameters assessed

#### Volumetric and functional parameters

Three-dimensional LVEF (3D LVEF) has been cited as a fundamental functional outcome in all five studies. Two studies reported quantitative three-dimensional left ventricular volumetric parameters, including end-diastolic volume (EDV), end-systolic volume (ESV), and stroke volume (reported in one study) [25], as well as indexed volumetric variables such as left ventricular end-diastolic volume index (LVEDVi) and left ventricular end-systolic volume index (LVESVi) (reported in one study) [28]. All available studies did not include systematic evaluation of the right ventricular functionality through 3D.

#### Three-dimensional strain and deformation parameters

The assessment of myocardial deformation in three dimensions was a key part of most of the studies included. Cruz and Guan and Karakulak concurrently evaluated global longitudinal strain (GLS), global circumferential strain (GCS), and global area strain (GAS) [23, 25, 28]. Esmaeilzadeh et al. compared parameters of strain on a global basis, but they did not conduct a segmental or regional analysis of strain [27]. Lambert et al. did not directly measure three-dimensional strain but determined 2D echocardiographic strain and CMR measures of deformations [26]. In Cruz et al. and Guan et al., regional strain analysis was performed with a 17-segment model of the left ventricles and a whole model of the left ventricles, respectively, and made it possible to evaluate the patterns of myocardial involvement spatially [23, 25]. The rest of the studies did not show regional strain evaluation.

#### Mechanical dyssynchrony and advanced metrics

Assessment of mechanical dyssynchrony and rotational mechanics was limited in the evidence base. No study reported three-dimensional indices of dyssynchrony or echocardiography-based rotational mechanics. Myocardial work indices, including global work index (GWI) and global work efficiency (GWE), were reported only by Guan et al. [23]. Esmaeilzadeh et al. assessed the additive value of cardiac biomarkers (high-sensitivity troponin I, BNP) in combination with imaging parameters but found no incremental diagnostic accuracy beyond imaging alone [27].

### Detection of cardiac dysfunction

#### Subclinical myocardial dysfunction

Across the five included studies, three-dimensional echocardiography was reported to be sensitive in identifying features suggestive of subclinical myocardial dysfunction which is usually shown by early impairment of myocardial strain parameters even when LVEF was preserved. Deterioration of GLS, GCS, and GAS was widespread in active cancer treatment and usually preceded 3D LVEF changes that were quantifiable. Cruz et al. and Guan et al. noted that the strain indices in the three cases showed a gradual declining nature at the initial stages of anthracycline-based chemotherapy combined with targeted therapy, with abnormalities in strain parameters being observed across treatment cycles prior to clinically overt cardiotoxicity [23, 25]. Equally, the research of Karakulak et al. identified a lack of deformation parameters in patients undergoing long-term TKI therapy regardless of LVEF, which gathers the importance of three-dimensional strain as an early sign of myocardial damage when anthracyclines are not administered [28].

#### Overt cardiac dysfunction

Overt cardiac dysfunction was determined by the decrease of LVEF but the levels of decrease varied among the studies based on guidelines or imaging modalities applied. CTRCD in longitudinal cohorts was characterized by a decrease in 3D LVEF, which is usually accompanied by a deterioration in strain parameters. The study by Esmaeilzadeh et al. showed that the 3D LVEF and global strain indexes combination had a higher diagnostic accuracy compared to CMR used as a reference standard [27]. Lambert et al. have found that 3D LVEF changed significantly over time in the patients who were subjected to sequential anthracycline and trastuzumab therapy [26].

## Discussion

### Findings from included studies

The present scoping review found the five observational studies that assessed the use of the 3DE to measure myocardial performance in cancer patients undergoing anthracycline-based chemotherapy and/or targeted therapy. The four prospective cohort studies and one cross-sectional study included in the study mainly included adult female patients with breast cancer in their late fifties and early forties with a controlled baseline of cardiovascular risk factors. Four studies were done to compare patients undergoing anthracycline-based chemotherapy combined with HER2-targeted therapy, and one study compared patients undergoing long-term TKI therapy without anthracycline exposure. In all the studies, 3DE was reported to be feasible in cardiac surveillance across included studies, and three-dimensional strain parameters were consistently associated with the identification of subclinical myocardial dysfunction prior to measurable declines in LVEF.

### Contextualization with broader literature

The discussion that follows is used to contextualize the results of the included studies in the context of the overall cardio-oncology literature. It should also be noted that the comparator studies mentioned below were not included in the evidence base and are mentioned to give relevant clinical and methodological background.

The study designs and demographic characteristics of the included studies are consistent with several comparator studies in literature. Santoro et al., Arciniegas Calle et al., Alam et al., Piveta et al., and El-Sherbeny et al. also employed prospective cohort designs in breast cancer patients receiving anthracycline-based therapy in combination with trastuzumab, with a focus on the early detection of subclinical myocardial dysfunction [24, 29–32]. These comparator studies have a sample size of between 50 and 136 patients which is comparable with those used in the present review. Nevertheless, not all of the comparator studies restricted to breast cancer patients: Wang et al. offered patients with diffuse large B-cell lymphoma [33], and Shen et al. provided broader oncology populations exposed to anthracyclines [34], which suggests that 3DE can be used across different types of cancer not well represented in the current review.

Regarding the relative performance of imaging modalities, the studies and the literature included has some information to offer to the clinical practice. Two-dimensional GLS is the most commonly employed parameter in the detection of subclinical dysfunction and it is recommended by recent guidelines as a first level surveillance tool due to its easy access and previously established prognostic significance [16]. TMAD provides an easy and reproducible longitudinal ventricular functional measurement but fails to measure circumferential or area mechanics. The theoretical benefit of 3D strain parameters is that multidirectional measurement of the acquisition is simultaneous, and the research in this review indicates that 3D GLS, GCS, and GAS have been reported to detect subclinical changes that cannot be detected by 2D methods alone. There is a dearth of head-to-head comparisons of 2D GLS, TMAD, and 3D strain parameters in identical populations of patients and the added clinical value of 3DE to existing 2D parameters is not clearly shown.

As far as CMR-based studies are concerned, Esmaeilzadeh et al. and Lambert et al. adopted CMR as a reference standard and have shown that 3D LVEF and global strain indices used in combination can give diagnostic quality equal or better than CMR-based parameters used to detect CTRCD [26, 27]. Though CMR is the best in terms of tissue characterization and is regarded as the gold standard of volumetric examination, its high cost, time requirements, and scarcity render it impossible to use as a regular surveillance method in most clinical scenarios [17]. These results suggest that 3DE may have potential as an alternative or complementary modality to CMR in serial monitoring; however, this requires confirmation in larger, standardised studies. Some comparator studies have captured areas of cardiotoxicity that have not been systematically assessed in the studies included. Zhao et al., El-Sherbeny et al., and Wang et al. assessed right ventricular dysfunction using 3DE, and the authors reported a change in the early right ventricular ejection fraction and strain as an indication of anthracycline-related cardiac injury [32, 33, 35]. The left ventricular pre-eminence of studies contained in this review can thus be a potential underestimation of the magnitude of biventricular implication that is becoming more apparent in the anthracycline-related myocardial injury.

The cumulative anthracycline doses in the studies included (in the range of 240 to 300 mg/m 2 doxorubicin equivalent) fall within the range of cumulative anthracycline doses in comparator studies [36, 37]. Nevertheless, there was inconsistency in the quantitative reporting of cumulative anthracycline dosing in both the included and comparator literature which impeded comparative interpretation [38].

Such advanced functional parameters as myocardial work indices (GWI, GWE) were measured in Guan et al. [23] and were less frequently measured in comparator studies, indicating that this is a relatively new field of research. The use of 3DE with CMR, found by Lambert et al. and Esmaeilzadeh et al. [26, 27], is an indication of a trend towards multimodality imaging validation in the cardio-oncology study.

## Limitations

Multiple shortcomings are to be taken into account when drawing conclusions about the findings of this review. To begin with, the number of observational studies was five only, and there was a heterogeneity in the study designs, populations, imaging protocols, regimens, and outcome definitions, which narrows the generalizability of the results. The search strategy was based on mostly title and abstract field of free-text search, a factor that might have compromised the sensitivity of the search. Studies in which three-dimensional echocardiography (3DE) analysis was reported only within the methods section, those using alternative terminology to describe cardiotoxicity, or those referring to mixed treatment regimens without explicitly specifying combination therapy within searchable fields may have been overlooked. This limitation is particularly relevant given the small number of studies included and suggests that the available evidence base may be underestimated. Additionally, individual study quality was not formally assessed, in accordance with Joanna Briggs Institute (JBI) recommendations for scoping reviews, which implies that the methodological rigour of the included studies cannot be fully ascertained. The populations found in breast cancer and the use of the HER2-targeted therapy make the results less applicable to other forms of cancer and classes of targeted therapies. Deduplication caused the elimination of 83 out of 108 records (76.9%); this percentage is high, although it is in agreement with the overlap between the five databases searched, and the precision of the final list of unique records (*n* = 25) was confirmed by manual verification.

## Conclusion

The available evidence suggests that three-dimensional echocardiography, particularly strain-based analysis, may have potential to facilitate earlier detection of subclinical myocardial dysfunction in patients receiving anthracycline-based chemotherapy and/or targeted therapy. Three-dimensional strain parameters appear to offer a practical advantage over conventional two-dimensional measures by enabling simultaneous multidirectional assessment, although their incremental clinical benefit over established parameters such as 2D GLS requires further investigation. Given the limited number of included studies and the methodological heterogeneity observed, these findings should be interpreted with caution and are best regarded as mapping the current state of evidence rather than supporting definitive clinical recommendations. Larger, standardised, multicentre prospective studies are necessary to establish the routine clinical role of 3DE in cardio-oncology surveillance and to inform its integration into cancer therapy cardiac monitoring guidelines.

## Supplementary Information

Supplementary Material 1.

## Data Availability

All data analysed in this study were derived from previously published studies included in the review. Extracted data tables are available from the corresponding author upon reasonable request.
