# Supplementary material for: Assessment of myocardial performance using three-dimensional echocardiography in cancer patients exposed to anthracycline-based chemotherapy and/or targeted therapy – a scoping review
Source: BMC Cardiovasc Disord. 2026 May 16;26:673. doi: 10.1186/s12872-026-05891-w (PMC13450073; doi:10.1186/s12872-026-05891-w)
Supplement: Supplementary file 1 [file 12872_2026_5891_MOESM1_ESM.docx]

**Supplementary Table S1. Database Search Strategies**

**A. MEDLINE (PubMed) Search Strategy**

| **Search ID** | **PCC Element** | **Search Strategy** | **Records** |
| --- | --- | --- | --- |
| #1 | Population – Cancer patients | (cancer*[Title/Abstract] OR oncology[Title/Abstract] OR neoplasm*[Title/Abstract] OR malignant*[Title/Abstract] OR tumor*[Title/Abstract] OR tumour*[Title/Abstract]) | 4,295,249 |
| #2 | Concept – Cancer therapy | ("anthracycline"[Title/Abstract] OR doxorubicin[Title/Abstract] OR epirubicin[Title/Abstract] OR daunorubicin[Title/Abstract] OR idarubicin[Title/Abstract] OR "targeted therapy"[Title/Abstract] OR "HER2 inhibitor"[Title/Abstract] OR trastuzumab[Title/Abstract] OR pertuzumab[Title/Abstract] OR "tyrosine kinase inhibitor"[Title/Abstract] OR TKI[Title/Abstract] OR "immune checkpoint inhibitor"[Title/Abstract] OR immunotherapy[Title/Abstract]) | 353,601 |
| #3 | Concept – 3D Echo | ("three-dimensional echocardiography"[Title/Abstract] OR "3D echocardiography"[Title/Abstract] OR 3DE[Title/Abstract] OR "three dimensional speckle tracking"[Title/Abstract] OR "3D speckle tracking"[Title/Abstract]) | 4,887 |
| #4 | Concept – Cardiotoxicity | ("myocardial function"[Title/Abstract] OR "cardiac function"[Title/Abstract] OR "myocardial performance"[Title/Abstract] OR cardiotoxicity[Title/Abstract] OR "cancer therapy-related cardiac dysfunction"[Title/Abstract] OR CTRCD[Title/Abstract]) | 77,054 |
| #5 | Combined | #1 AND #2 AND #3 AND #4 | 46 |

**B. Embase Search Strategy**

| **Search ID** | **Search Strategy** | **Records** |
| --- | --- | --- |
| #1 | ('cancer'/exp OR cancer:ti,ab,kw OR oncology:ti,ab,kw OR neoplasm*:ti,ab,kw OR malignan*:ti,ab,kw) | 1,123 |
| #2 | ('anthracycline'/exp OR anthracycline:ti,ab,kw OR doxorubicin:ti,ab,kw OR epirubicin:ti,ab,kw OR daunorubicin:ti,ab,kw OR idarubicin:ti,ab,kw OR 'targeted therapy'/exp OR 'her2 inhibitor':ti,ab,kw OR trastuzumab:ti,ab,kw OR pertuzumab:ti,ab,kw OR 'tyrosine kinase inhibitor'/exp OR tki:ti,ab,kw OR 'immune checkpoint inhibitor'/exp OR immunotherapy:ti,ab,kw) | 565 |
| #3 | ('three dimensional echocardiography'/exp OR '3d echocardiography':ti,ab,kw OR 3de:ti,ab,kw OR 'three dimensional speckle tracking':ti,ab,kw OR '3d speckle tracking':ti,ab,kw) | 1,285 |
| #4 | ('myocardial function'/exp OR 'cardiac function':ti,ab,kw OR cardiotoxicity:ti,ab,kw OR 'cancer therapy related cardiac dysfunction':ti,ab,kw OR ctrcd:ti,ab,kw) | 3,331 |
| #5 | #1 AND #2 AND #3 AND #4 | 23 |

**C. Scopus Search Strategy**

| **Search ID** | **Search Strategy (TITLE-ABS-KEY)** | **Records** |
| --- | --- | --- |
| #1 | (cancer OR oncology OR neoplasm* OR malignan*) | 7,877 |
| #2 | (anthracycline OR doxorubicin OR epirubicin OR daunorubicin OR idarubicin OR "targeted therapy" OR "HER2 inhibitor" OR trastuzumab OR pertuzumab OR "tyrosine kinase inhibitor" OR TKI OR "immune checkpoint inhibitor" OR immunotherapy) | 677 |
| #3 | ("three-dimensional echocardiography" OR "3D echocardiography" OR 3DE OR "three-dimensional speckle tracking" OR "3D speckle tracking") | 459 |
| #4 | ("myocardial function" OR "cardiac function" OR cardiotoxicity OR "cancer therapy-related cardiac dysfunction" OR CTRCD) | 654 |
| #5 | #1 AND #2 AND #3 AND #4 | 28 |

**D. Web of Science Search Strategy**

| **Search ID** | **Search Strategy (TS = Topic Search)** | **Records** |
| --- | --- | --- |
| #1 | TS = (cancer OR oncology OR neoplasm* OR malignan*) | 345 |
| #2 | TS = (anthracycline OR doxorubicin OR epirubicin OR daunorubicin OR idarubicin OR "targeted therapy" OR "HER2 inhibitor" OR trastuzumab OR pertuzumab OR "tyrosine kinase inhibitor" OR "immune checkpoint inhibitor" OR immunotherapy) | 213 |
| #3 | TS = ("three-dimensional echocardiography" OR "3D echocardiography" OR 3DE OR "three-dimensional speckle tracking" OR "3D speckle tracking") | 562 |
| #4 | TS = ("myocardial function" OR "cardiac function" OR cardiotoxicity OR "cancer therapy-related cardiac dysfunction" OR CTRCD) | 110 |
| #5 | #1 AND #2 AND #3 AND #4 | 11 |

**E. Cochrane Library (CENTRAL) Search Strategy**

| **Search ID** | **Search Strategy** | **Records** |
| --- | --- | --- |
| #1 | ("three-dimensional echocardiography" OR "3D echocardiography" OR 3DE OR "three-dimensional speckle tracking" OR "3D speckle tracking") AND (cancer OR oncology OR malignan*) AND (anthracycline OR doxorubicin OR epirubicin OR daunorubicin OR idarubicin OR "targeted therapy" OR "HER2 inhibitor" OR trastuzumab OR pertuzumab OR "tyrosine kinase inhibitor" OR "immune checkpoint inhibitor" OR immunotherapy) | 21 |
| #2 | ("myocardial function" OR "cardiac function" OR cardiotoxicity OR "cancer therapy-related cardiac dysfunction" OR CTRCD) | 17 |
| #3 | #1 AND #2 | 0 |

*Note: Searches were conducted from database inception to November 2025. Controlled vocabulary (MeSH terms in MEDLINE, Emtree terms in Embase) were used where available in addition to free-text terms. The decision to search primarily in title and abstract fields was made to balance specificity with feasibility, as discussed in the Methodology section.*
